# Impact of mandibular 4-implant overdenture base construction techniques on assessment of occlusion with digital occlusion analysis system (clinical crossover study)

**DOI:** 10.1186/s12903-025-06228-3

**Published:** 2025-05-30

**Authors:** Ahmed Ali Abdelghany, Mohammed M. Fouad, Radwa Mohsen Kamal Emera, Osama Askar, Ayman Ahmed Mustafa Yaseen

**Affiliations:** 1 Department of Prosthodontics, Faculty of Dentistry, Mansoura University, Mansoura, Egypt; 2 OMFS Department, Faculty of Dentistry, Mansoura University, Mansoura, Egypt

**Keywords:** CAD/CAM milled denture base, OccluSense, Conventional denture base, Overdenture, Occlusal contact stability

## Abstract

**Background:**

Occlusion plays a crucial role in the long-term success and prognosis of implant-supported overdentures. The method used to fabricate the overdenture base, whether conventional or CAD/CAM milled, could influence occlusal contact balance. However, definitive evidence on this matter remains lacking. Thus, this study aimed to compare two fabrication techniques, CAD/CAM milled and conventional, for four-implant-supported complete mandibular overdenture bases, with a specific focus on their impact on occlusal balance.

**Methods:**

Edentulous patients participated in this study received four-implant supported mandibular overdentures constructed using two different types of overdenture bases: CAD/CAM milled and conventional bases. A total of 21 patients, representing 42 overdentures, were enrolled in the study. Occlusal adjustments were made for each overdenture after picking up of attachments. The patients were classified randomly and equally into two groups: **Group I**: patients delivered maxillary complete dentures opposed to four implant-supported mandibular overdentures constructed with CAD/CAM milling followed by conventionally constructed dentures. **Group II**: patients delivered maxillary complete dentures opposed to four implant-supported mandibular overdentures constructed with conventional method followed by CAD/CAM milled dentures. According to the type of denture bases, dentures were classified into two equal groups: **Group A**: CAD/CAM constructed overdenture bases. **Group B**: conventionally constructed overdenture bases. For each overdenture group, occlusal analysis measurements were recorded at overdenture delivery (T_0_) and after three months of denture wearing (T_3_). Data was analyzed using the Statistical Package of Social Science (SPSS) program. Repeated measures ANOVA were used to test significant differences in occlusal force distribution between observation intervals, groups and locations followed by Bonferroni post hoc test for multiple comparisons. Independent samples t-test was used to compare occlusal force between groups. P is significant if < 0.05 at confidence interval 95%.

**Results:**

Comparing different occlusal contact locations in each group at (T_0_) showed a significant difference between anterior and posterior locations whereas comparing different occlusal contact locations in each group at (T_3_) showed a significant difference between molar and premolar locations for group B while insignificant between molar and premolar locations for group A. The comparison between different intervals within group A revealed insignificant differences while significant occlusal changes at premolar and molar regions were presented within group B.

**Conclusions:**

The four implant-supported CAD/CAM milled overdenture bases offer greater advantages over conventional ones in terms of occlusal contact stability.

**Clinical Trial Registry Number:**

(NCT06080815))08/10/2023).

## Background

Mandibular complete dentures often struggle with insufficient retention and stability, potentially impacting patient satisfaction and comfort. Completely edentulous patients could be rehabilitated with overdentures enhanced with at least two to four implants. Studies with long-term follow-ups indicate that using four implants yields superior results and optimal survival rates. This approach offers greater stability during function, minimizing movement and excessive loading that could jeopardize osseointegration [1]. 

The traditional method of complete dentures construction is intricate and challenging for both dentists and dental technicians. It also demands multiple patient visits and extensive laboratory work. Moreover, heat-cured acrylic resins fall short of meeting all the criteria for an ideally perfect denture base material [2]. 

The integration of computer-aided design/computer-aided manufacturing (CAD/CAM) techniques into the fabrication of complete dentures has resulted in the evolution of modified and easier clinical protocols, the use of materials with enhanced properties, and improved fit and retention of the dentures. This advancement has also shortened chairside and laboratory times, leading to a decrease in overall clinical and laboratory workflow. Reports indicate high levels of satisfaction among both patients and clinicians regarding CAD/CAM complete dentures [3]. 

The CAD/CAM clinical protocols are evolving quickly, with new alternative methods integrating traditional clinical steps designed to meet the necessary criteria for successful CAD/CAM complete denture fabrication. While efforts to combine optical scans with conventional clinical procedures have shown some promise, a fully digital workflow for complete denture fabrication has not yet been established [4–6]. 

The prepolymerized acrylic resin employed in the production of digital denture bases offers enhanced fit and strength compared to traditional bases. Unlike conventionally processed materials, the milled prepolymerized acrylic resin does not experience polymerization shrinkage. Research shows that prepolymerized acrylic resin (PAR) has lower residual monomer content and is more hydrophobic than its conventional counterparts. This characteristic helps decrease the risk of infections, as fewer microorganisms, such as Candida albicans, adhere to the denture bases [7]. 

Conventional resins typically exhibit a lower modulus of elasticity compared to CAD/CAM resins, likely due to differences in their manufacturing processes. This modulus is influenced by the residual monomer content; higher monomer levels tend to lower the glass transition temperature, resulting in increased flexibility of the resin. Resins with a greater modulus of elasticity are better at resisting elastic deformation, contributing to more stable occlusion [8]. 

Achieving occlusal equalization in removable complete denture prostheses is a primary objective for prosthodontists. Modern technology provides a digital solution to this challenge, known as OccluSense. This tool enables clinicians to make computer-guided adjustments for occlusal force, resulting in measurable improvements in the balance of the installed prosthetic occlusion [9]. Utilizing this technology, clinicians can easily assess the distribution of occlusal forces and identify specific adjustments needed to achieve a centered and balanced force distribution. The goal of these adjustments is to align the occlusal forces along the midline of the hard palate, achieving an almost equal balance of 50% on the right and 50% on the left. This balanced distribution enhances the denture’s tissue seating, ensuring that during chewing, the occlusal forces are transmitted to the broadest and most supportive areas of the tissue [10]. 

Occlusion plays a crucial role in the long-term success and prognosis of implant-supported overdentures. The method used to fabricate the overdenture base, whether conventional or CAD/CAM milled, could influence occlusal contact balance. However, definitive evidence on this matter remains lacking. Thus, this study aimed to compare two fabrication techniques, CAD/CAM milled and conventional, for four-implant-supported complete mandibular overdenture bases, with a specific focus on their impact on occlusal balance.

## Methods

Patients for this cross-over study (aged between 55 and 65 years) were selected from the Clinic of Prosthodontic Department, Faculty of Dentistry, Mansoura University seeking prosthetic rehabilitation. All patients were informed about the line of treatment and follow up recalls during surgical and prosthodontic procedures. Any failure of implants will be replaced by another suitable prosthodontic treatment.

Patients in this study were selected based on specific criteria. All participants had completely edentulous alveolar ridges with healthy, firm mucosa, free from inflammation. They also had sufficient quality and quantity of the mandibular residual alveolar ridge for implants, confirmed by cone beam computed tomography (CBCT). All participants exhibited an Angle’s Class I maxillomandibular relationship with an interarch space of at least 10–12 mm. Additionally, they were in good health, free from systemic diseases affecting bone health, such as uncontrolled diabetes and osteoporosis. Those with a history of parafunctional habits, smoking, alcoholism, surgical contraindications, or prior radiation therapy in the head and neck were excluded from the study.

The sample size of this study was calculated on the G-power program (version 3.1.9.7). We conducted the sample size calculation using a medium effect size as the basis for our statistical analysis. Assuming a medium effect size (f) of 0.25, an acceptable error margin of 5% (d = 0.05), and a desired statistical power of 80%, the study involves two groups of participants to compare significant differences in occlusal force distribution across observation times (using repeated measures ANOVA). The minimum sample size required for this study is 34 subjects. To account for potential missing data during follow-up, we included an additional 8 participants, bringing the total sample size to 42. The sample size for this study was similar to that used in a recent study [11] with a comparable design, the minimum sample size required to test significant differences in the percentage of occlusal contact between the two groups of participants is 40. This research was conducted in accordance with the Declaration of Helsinki. Ethical approval for the study was granted by the Institutional Ethics Committee of the Faculty of Dentistry, Mansoura University, Egypt (Approval No. 05201021). Informed consent was obtained from all participants prior to treatment.

**Fig. 1:**
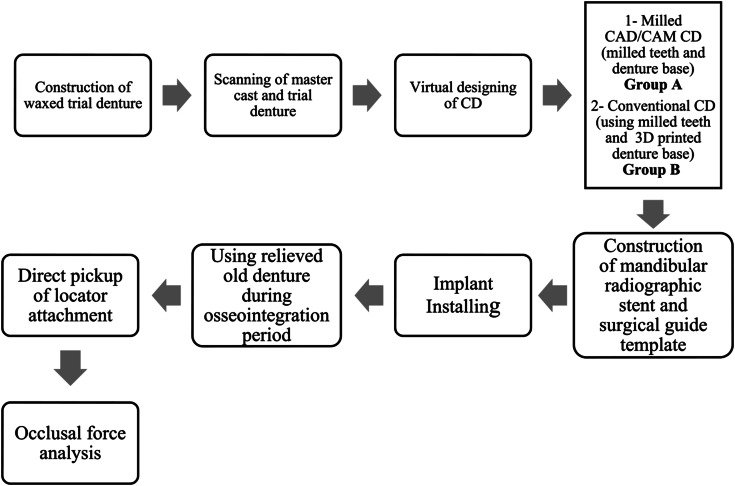
Flowchart representing prosthetic and surgical steps of the research

The prosthetic and surgical steps were done as follows (Fig. 1):

I. Construction of waxed trial denture (for group A and B): preliminary impressions were made with irreversible hydrocolloid impression material (Alginate impression material Cavex CA37, Cavex) after that, impressions were poured in stone (Dental stone, Super-cal IV, COE Laboratories Inc.) to obtain study casts over which customized trays (Self-cured acrylic resin, Acrostone) were fabricated for final impression making using zinc oxide impression material (Zinc oxide eugenol-free impression material, Cavex Outline). Jaw relations were recorded, and maxillary cast was mounted on semi adjustable articulator (A7 plus articulator, Bio-Art Equipamentos odotologicos LTDA) using facebow (Elite face-bow, Bio-Art Equipamentos odotologicos LTDA) then centric interocclusal record was used for mounting mandibular cast. Also, protrusive condylar angles were recorded, and lateral condylar angles were calculated from Hanau formula. Acrylic semi-anatomic teeth (VITA Zahnfabrik H. Rauter GmbH & Co. KG) were selected and modified to be arranged following the medially positioned lingualized occlusion. The maxillary and mandibular trial dentures were tried in the patient’s mouth and verified for occlusal plane orientation, vertical dimension, and horizontal relations.

II. Construction of CAD/CAM dentures following Geneva protocol [3] (group A): For all patients, master casts together with the mounted trial dentures were lightly coated with antiglare spray (D-Scan, Dentify GmbH) and scanned with Desktop 3D Scanner (Shining 3D Autoscan-DS-EX) and the resultant scan data were stored in standard tessellation language (STL) file format. The scanned data was imported into a design software program (exocad DentalDB 3.2Elefsina 8820), after virtual designing of complete denture, data was transferred to CAM software program (MillBox DGSHAPE Edition v3.7.3) for milling two sheets of the artificial teeth (generic denture S for Anteriors and generic denture S Lingualized for Posteriors) and the denture base for the digital prosthesis. The intaglio of the milled teeth and bonding surface of the denture bases were air particles abraded with 50 μm aluminum oxide for 5 s each at 20 Psi. Milled teeth were then bonded to a denture base with a CAD bonding agent (Ivoclar Ivobase CAD bonding, Ivoclar Vivadent). The CAD/CAM milled denture was finished and polished. Digital denture was inserted, and clinical remounting was done to correct any errors resulting from resiliency of oral mucosa. Follow up of the patients one day and one week after denture insertion for any post insertion complaint.

III. Construction of conventional dentures utilizing the milled sheets of artificial teeth (group B): Utilizing STL file for each patient, trial base was 3D printed, and the other sheet of milled teeth was bonded by wax to this denture base guided by their sockets on the semi-adjustable articulator. Trial denture was processed by conventional flasking method to produce heat-cured acrylic resin denture. The conventional denture was finished and polished. After that, it was inserted, and clinical remounting was done to correct any errors resulting from resiliency of oral mucosa. The dentures were delivered after clinical adjustments, giving post insertion instructions along with denture hygiene and maintenance information to the patient.

IV. Construction of mandibular radiographic stent and surgical guide template: To select the prospective implant sites, the old mandibular denture of each patient was duplicated from clear acrylic and modified with gutta-percha radio-opaque markers to be used as a radiographic stent. Following dual scan protocol, a mucosal supported Stereolithographic surgical guide from 3D printer was constructed and used for implant placement.

**Fig. 2:**
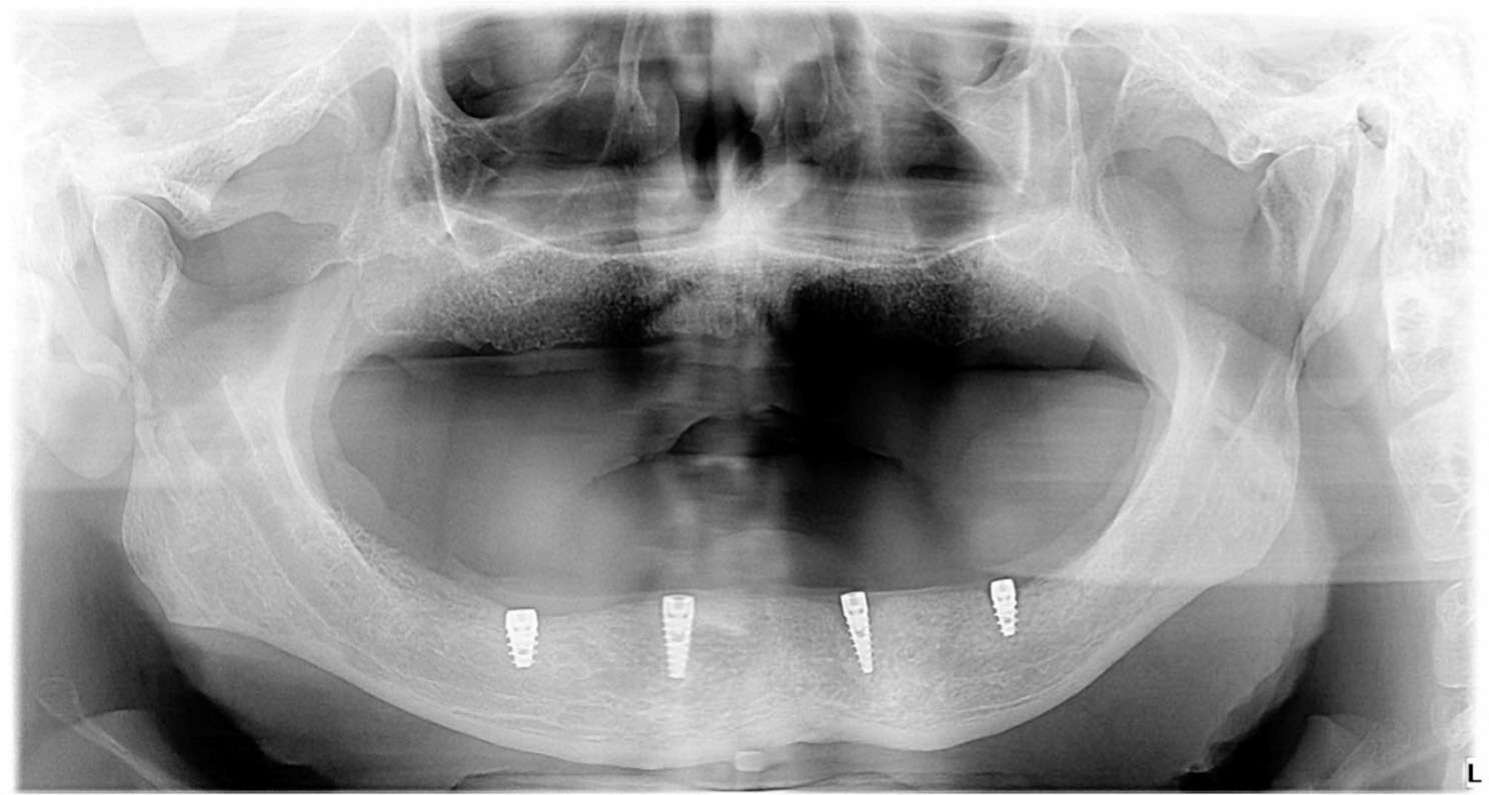
Panoramic x-ray after implant insertion

V. Implant installation: For every patient, two axial implants (J Dental Care Implant System) were surgically inserted in the canine areas and two axial implants were inserted in the 1st molar regions. (Fig. 2) After radiographic verification of implants location, low profile healing abutments were screwed in the internal hex of the implants using hex key. The old mandibular denture fitting surface was relieved opposed to healing abutments to be used during the osseointegration period. Implants were left unloaded for 3 months according to the delayed loading protocol. All patients were limited to a soft diet for 10 days.

**Fig. 3:**
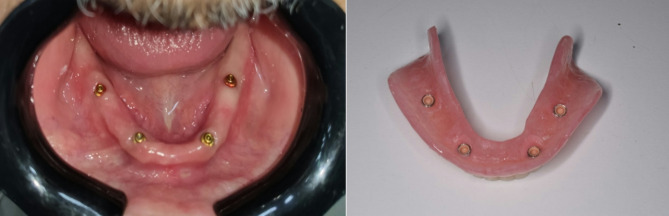
Screwed locator attachments and picked up of four metal housings

VI. Locator attachments were picked up directly with self-cure acrylic resin in the two groups of the study dentures. (Fig. 3)

**Fig. 4:**
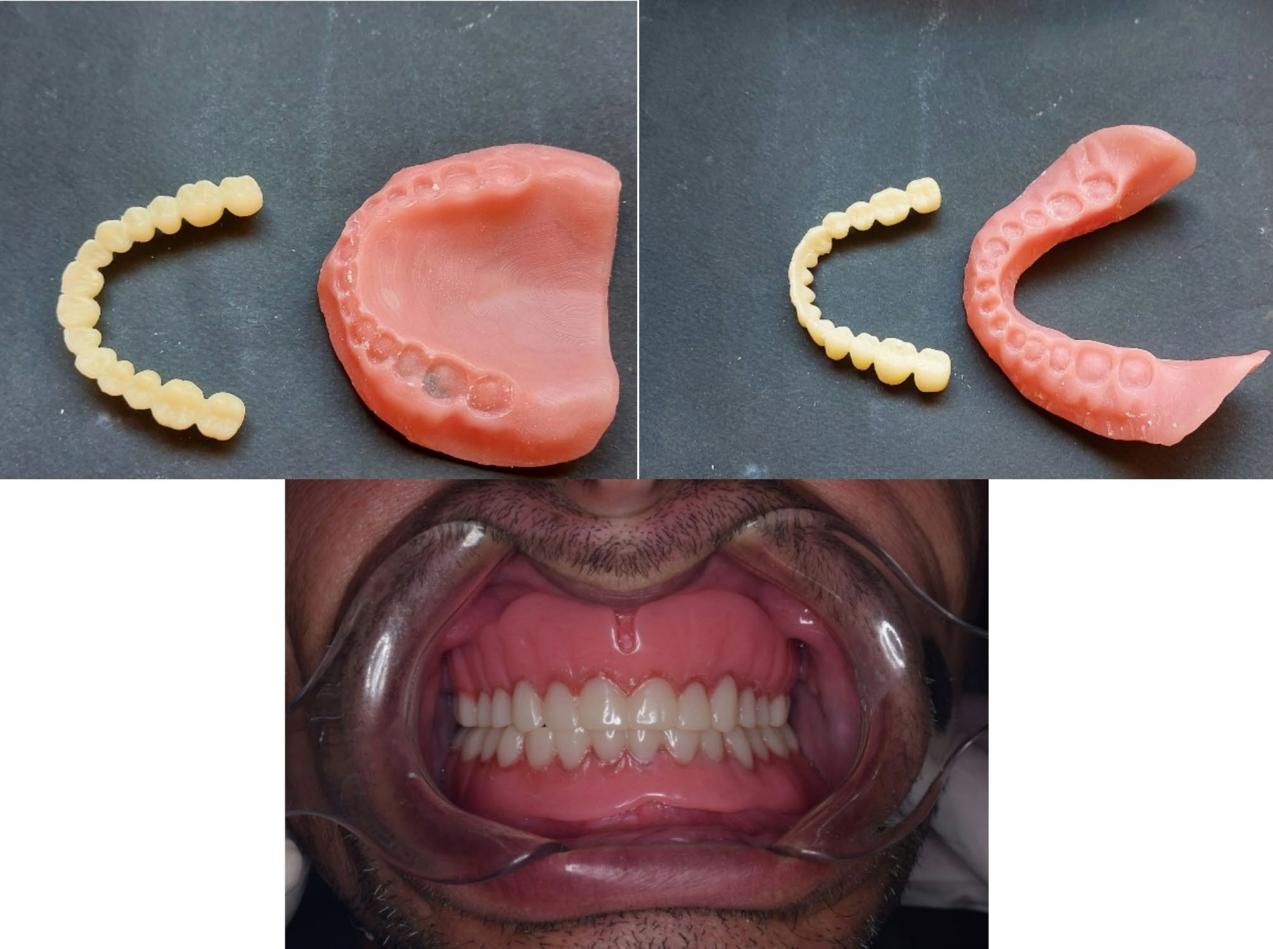
(Group A) Milled denture base and artificial teeth

**Fig. 5:**
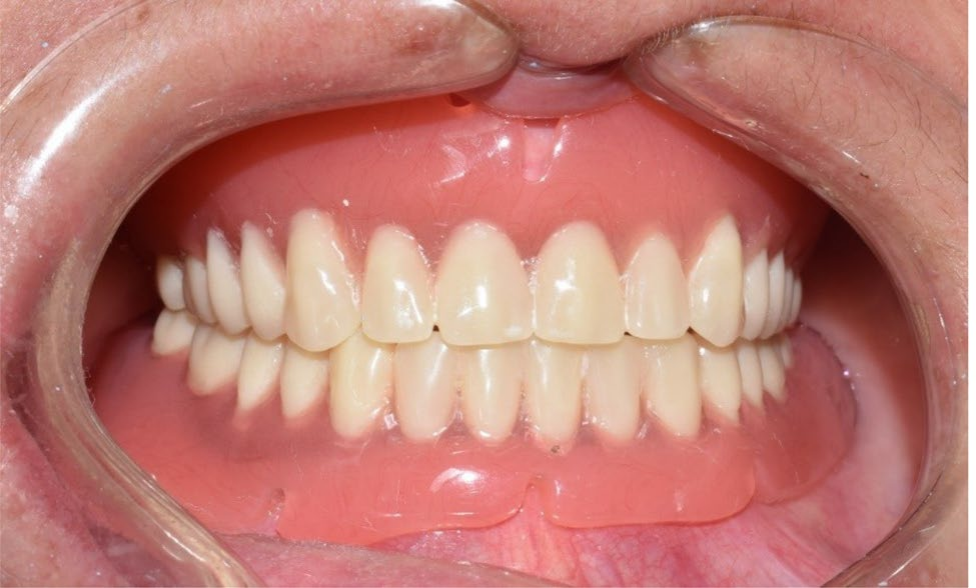
(Group B) Conventionally constructed denture base and milled artificial teeth

*Patients and dentures grouping*, patients were classified randomly and equally into two groups: **Group I**: patients received maxillary complete dentures opposed to four implant-supported mandibular overdentures constructed with CAD/CAM milling followed by conventionally constructed dentures. **Group II**: patients received maxillary complete dentures opposed to four implant-supported mandibular overdentures constructed with conventional method followed by digitally constructed dentures. According to the construction techniques of denture bases, dentures were classified into two equal groups: **Group A**: CAD/CAM constructed denture bases (Fig. 4). **Group B**: conventionally constructed denture bases (Fig. 5).

### Occlusal force assessment

**Fig. 6:**
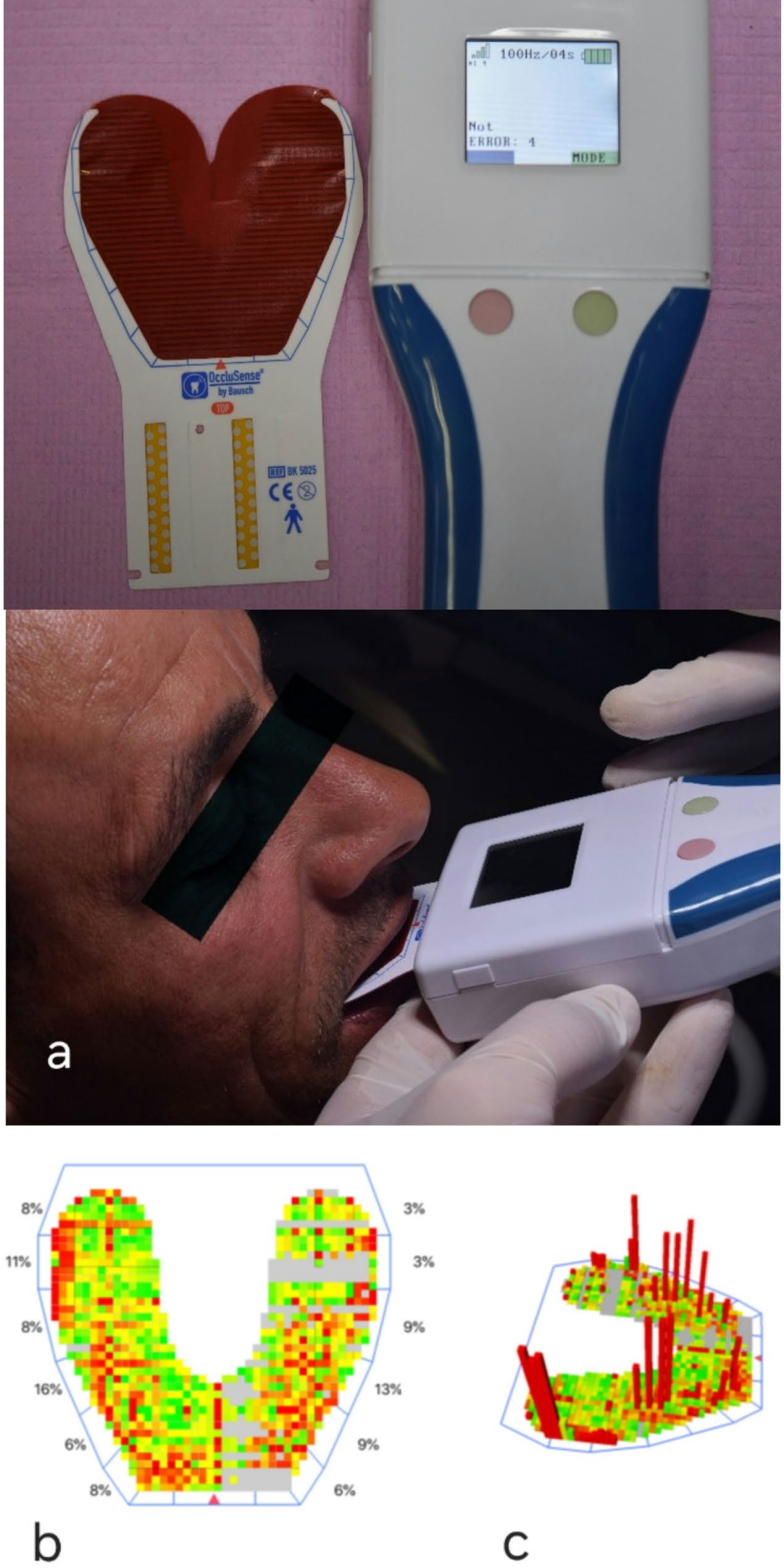
Digital analysis of occlusal forces utilizing OccluSense

After the installation of OccluSense (Bausch, Köln), the patient asked to open his mouth and the OccluSense sensor was inserted with the arrow (red triangle on the sensor midline) placed between the central incisors then asked to bite on the sensor (Fig. 6a). Relative pressure gradients are illustrated in the 2D Force Snapshot window (Fig. 6b) using neighboring data blocks in green, yellow, orange, and red, with their colors changing according to the pressure distribution across different areas of the arch. Contacts with small surface areas (pinpoint contacts) are indicated by orange and red, while larger surface area contacts (broad contacts) are represented by green and yellow. In the 3D rotational window (Fig. 6c), these colored blocks appear as columns of varying heights. By analyzing the recorded force movie frame by frame, high-pressure points can be adjusted sequentially based on the digital force scan of occlusal contact timing data. The goal is to equalize the force percentage between the right and left arch halves and ensure a nearly equal distribution of force across each posterior tooth counterpart, while maintaining light contacts in the anterior region.

For group (I) of patients, CAD/CAM dentures were delivered first and used for three months followed by two weeks of resting period, then conventional dentures were delivered for the other three months. For group (II) of patients, conventional dentures were delivered first and used for three months followed by two weeks of resting period, then CAD/CAM dentures were delivered for the other three months.

For each overdenture group, occlusal analysis measurements were recorded after picking up of attachment followed by equalization of occlusal forces by selective grinding (T0). After three months of denture wearing, occlusal analysis measurements were recorded (T3).

### Statistical analysis

The SPSS statistical package for social science version 25 (SPSS Inc., Chicago, IL, USA) was used for data analysis. Shapiro-Wilk test was used to test the normality of the recoded retention values. The data was parametric and normally distributed. Descriptive statistics were performed in terms of mean, standard deviation. Repeated measures ANOVA were used to test significant differences in occlusal force distribution between observation times/time intervals, groups and locations followed by Bonferroni post hoc test for multiple comparisons. Independent samples t-test was used to compare occlusal force between groups. P is significant if < 0.05 at confidence interval 95%.

## Results

OccluSense shows average levels of occlusal forces in percentages (%) for each segment of the dentition, the arch is divided into right and left halves, each half is subdivided into anterior, premolar and molar regions.

A comparison between different occlusal contact locations in each group at time of insertion (T0) regarding the mean of occlusal analysis is presented in Table 1. There was a significant difference between anterior and posterior locations. For all groups, the highest occlusal force was noted at molar areas followed by premolar areas and the lowest occlusal force was noted at anterior area.

**Table 1:** Comparison between different locations in each group regarding the mean occlusal analysis (force distribution) at T_0_

Groups	Locations			One Way ANOVA *P* Value
	Anteriors	Premolars	Molars	
A	8.25a ± 2.71	19.25b ± 3.19	22.50b ± 3.42	< 0.001*
B	10.38a ± 3.92	18.75b ± 3.24	22.88b ± 2.90	< 0.001*

* p is significant at 5%. Different letters in the same raw indicate significant differences between positions. Similar letters indicate no significant difference between positions

A comparison of different occlusal contact locations in each group after 3 months (T3) regarding the mean of occlusal analysis is presented in Table 2. There was a significant difference between molar and premolar locations for group B while, insignificant between molar and premolar locations for group A.

**Table 2:** Comparison between different locations in each group regarding the mean occlusal analysis (force distribution) at T_3_

Groups	Locations			One Way ANOVA *P* Value
	Anteriors	Premolars	Molars	
A	7.00a ± 3.66	22.13b ± 8.00	25.88b ± 8.96	< 0.001*
B	10.00a ± 3.42	25.25b ± 6.18	18.75c ± 5.49	0.002*

* p is significant at 5%. Different letters in the same raw indicate significant differences between positions. Similar letters indicate no significant difference between positions

On the other hand, Comparison between different intervals within group A (CAD/CAM overdenture) regarding the mean occlusal analysis is presented in Table 3. It revealed insignificant differences between time intervals. While the comparison between different intervals within group B (Conventional overdenture) regarding occlusal analysis is presented in Table 4. It revealed significant occlusal changes at premolar and molar regions.

**Table 3:** Comparison between different intervals within group (A) regarding the mean occlusal analysis (force distribution) at different locations

Location	T_0_	T_3_	*P* value
Anterior	8.25 ± 2.71	7.00 ± 3.66	0.898
Premolar	19.25 ± 3.20	22.13 ± 9.31	0.785
Molar	22.50 ± 3.42	25.88 ± 8.97	0.159

* p is significant at 5%

**Table 4:** Comparison between the two intervals of study within group (B) regarding the mean occlusal analysis (force distribution) using occlusense at different locations

Location	T_0_	T_3_	*P* value
Anterior	10.38 ± 3.93	10.00 ± 3.42	0.874
Premolar	18.75 ± 4.14	25.25 ± 7.28	0.048*
Molar	22.88 ± 2.90	18.75 ± 5.50	0.049*

* p is significant at 5%

## Discussion

A crossover design was selected because it eliminates inter-subject response variation to the same treatment, because all treatments are applied to all participants. This increases the statistical efficiency of the study, given the need for a smaller number of patients. The main disadvantage of the crossover trial is that the effects of one treatment can linger and influence the response to later treatments. To mitigate this issue, a common strategy is to implement a washout period (during which no treatment is administered) between successive treatments, ensuring that this interval is long enough for the effects of the previous treatment to dissipate [12]. 

In this study, comparing different occlusal contact locations in each group at time of insertion (T0) regarding the mean of occlusal analysis showed that there was significant difference between anterior and posterior locations. For all groups, the highest occlusal force was noted at molar areas followed by premolar areas and the lowest occlusal force was noted at anterior area. This may occur because we evaluate the occlusal contacts after adjustments of occlusion by selective grinding in a stable centric relation where occlusal forces intended to be more at molar and premolar regions.

The possible clarifications are compatible with the force finishing protocol introduced by S. Koirala [13]. It is a clinical technique employed to obtain occlusal harmony in dentistry following certain steps. Firstly, the occlusion of all teeth was adjusted by selective contouring ensuring that anterior light contact was maintained. Then, measure tooth-contact forces. After that, equalize right and left arch-half force percentages and distribute nearly equal force percentage on each posterior tooth counterpart.

Moreover, comparing different occlusal contact locations in each group after 3 months (T3) regarding the mean of occlusal analysis showed that there was a significant difference between molar and premolar locations for group B while, insignificant between molar and premolar locations for group A. The significant changes in occlusion after three months of using a conventional heat-cure acrylic resin denture base, even when supported by canine and first molar implants, can be attributed to the alteration of denture base fit with time and deformation resulted from the inherent properties of the heat cured acrylic material, denture settlement, and the functional forces acting on the prosthesis. These factors collectively contribute to the instability of occlusal relationships over time. Consequently, the null hypothesis which stated that there are no differences between the two different construction techniques (CAD-CAM milled and conventional techniques) of four-implant assisted complete mandibular overdenture concerning the digital occlusal force analysis, was rejected.

Conventional heat-cure acrylic resins are known to undergo dimensional changes due to factors such as polymerization shrinkage, thermal expansion, and water absorption. These changes can lead to alterations in the fit of the denture base over time, affecting occlusion [14]. Acrylic resins can wear down or deform under functional loads, which may lead to changes in occlusal relationships. This wear can be exacerbated by the masticatory forces exerted during chewing. Conventional denture bases, typically made from heat-cured acrylic resin, may not possess the same level of flexural strength as milled or 3D-printed alternatives. This can lead to deformation under occlusal forces, which negatively impacts the occlusal relationship and can cause discomfort during function [8, 15]. 

On the other hand, Comparison between different intervals within group A (CAD/CAM OD) regarding the mean occlusal analysis revealed insignificant difference between time intervals. This may denote that the occlusal relation between maxillary and mandibular teeth remain stable which can be explained by the resistance of CAD/CAM milled denture bases to deformation as a result of their superior mechanical properties.

This explanation could be consistent with Aguirre [7] and Prpić [16]. They reported that CAD/CAM milled denture bases showed superior mechanical properties.

Primarily, CAD/CAM resins exhibit higher flexural strength, a key property that characterizes the material’s rigidity. This flexural strength is influenced by the degree of polymerization achieved during processing. The elevated flexural strength values of CAD/CAM resins can be attributed to a higher degree of conversion. These resins are milled from solid, prepolymerized blocks, which are polymerized using equipment that offers greater polymerization potential compared to conventional processing methods. As a result, the increased rigidity of milled resins leads to deformation-resistant materials that provide a more stable occlusion [7, 16]. 

Another explanation for this insignificant difference may be attributed to the more accuracy of CAD/CAM milled denture base with little dimensional changes resulting in more adaptation and less settlement.

Several studies have concluded that CAD/CAM milled denture bases were found to be the most accurate with high precision of fit when compared to pack and press fabrication techniques used with heat-cure acrylic resin as measured by surface matching software [14, 17–19]. 

While the comparison between different intervals within group B (Conventional OD) regarding occlusal analysis revealed significant occlusal changes at premolar and molar regions. This may indicate deformation in conventional denture bases due to its inferior mechanical properties that affect the occlusal relation between the posterior teeth.

This illustration could be agreed with Batisse, et al. [8] They noted that conventional denture bases exhibit inferior mechanical properties and reduced resistance to deformation. This decline in mechanical properties is likely a result of the manufacturing process. Lower modulus of elasticity and flexural strength are associated with higher residual monomer content. Research has demonstrated that elevated monomer levels lower the glass transition temperature, resulting in increased flexibility and decreased resistance to elastic deformation in the resin.

The potential limitations of this research include a relatively short study duration. Additionally, the study relies on a single evaluation method. We recommend prolonged study duration and using larger sample size.

## Conclusion

In view of the results of this clinical cross-over study, the four implant-supported CAD/CAM milled overdenture bases offer greater advantages over conventional ones in terms of occlusal contact stability.

## Data Availability

The datasets used in the current study are available from the corresponding author upon request.
